# Immunoasssay chromatographic antigen test for rapid diagnosis of Group A beta hemolytic Streptococcus pharyngitis in children: A cross/ sectional study

**Published:** 2011-06

**Authors:** S Noorbakhsh, A Tabatabaei, M Farhadi, Taj F Ebrahimi

**Affiliations:** 1 Pediatric Infectious Diseases Research center,Tehran University of Medical Sciences, Tehran, Iran; 2 Research Center for Diseases of Ear, Nose and Throat, Tehran University of Medical Sciences, Tehran, Iran

**Keywords:** Pharyngitis, Group A beta hemolytic Streptococcus (GABHS), Immunochromatograhic rapid test

## Abstract

**Background and Objective:**

Group A beta-hemolytic streptococcus (GABHS) is an important pharyngotonsillitis etiologic agent in children. The objective of this study was diagnosis of streptococcal pharyngitis based on rapid antigen detection test and conventional pharyngeal culture.

**Materials and Methods:**

The rapid GABHS antigen detection test was compared to culture on blood agar, the gold standard for the diagnosis of this etiologic agent.

**Results:**

Streptococcal antigen was detected in pharyngeal specimens of 34.5% of cases by rapid strip test. We detected group A Streptococcus in 17.2% of pharyngeal culture. There was no agreement between two methods ( PV < 0.1). The negative pharyngeal culture results are probably due to antibiotic usage in 43.2% of patients. Positive rapid test results in pharyngeal swab was age dependent ( P < 0.05). There was good correlation between observing the “petechia in pharynx of patients” and positive rapid test in pharyngeal swab (P < 0.004). Throat culture results were relatated to previous antibiotic usage ( P < 0.03).

**Conclusion:**

The rapid test in pharyngeal swab is helpful for rapid diagnosis and treatment of GABHS pharyngitis. Diagnosis of GABHS pharyngitis based on soley clinical findings is misleading in the majority of cases. Petechia observed in pharynx of the cases was highly predictive of streptococcal pharyngitis.

## INTRODUCTION

Group A beta-hemolytic streptococcus (GABHS) is an important pharyngotonsillitis etiologic agent (1, 2). However, clinical diagnostic methods are not reliable (3, 4). Correct etiologic diagnosis and early treatment is very important for preventing the suppurative and non-suppurative complications of streptococcal pharyngotonsillitis (5–7). Pharyngitis is a common disease in Iranian children (8). GABHS and *S. pneumonia* had been searched as causative of pharngitis of children in several studies (9–15). Rapid detection methods of GABHS antigen are useful to diagnose this agent (13–18). The aims of this study was diagnosis of streptococcal pharyngitis using the “Rapid antigen detection test” and conventional pharyngeal culture.

## MATERIALS AND METHODS

This cross sectional study had been done on 187 children (2–180 months; mean=64±48months) with fever and pharyngitis attended at Pediatrics Clinic in Rasoul Akram hospital and Shahid Heidari clinic (1387–1389)****. This study was approved by the Ethical Committee of Tehran University of Medical Sciences. Consent Letter obtained from parents.

Initially, a data collection form was completed by an authorized physician for each cases, followed by a complete clinical exam.

**Inclusion criteria.** acute onset of fever and pharyngitis

**Exclusion criteria.** Diagnosed cases with acute pharyngitis except GABHS included viral phatyngitis (influenza, adenovirus, RSV; confirmed with rapid tests), allergic pharyngitis, referral pain (otitis media, teeth infection), etc.

**Fig. 1:**
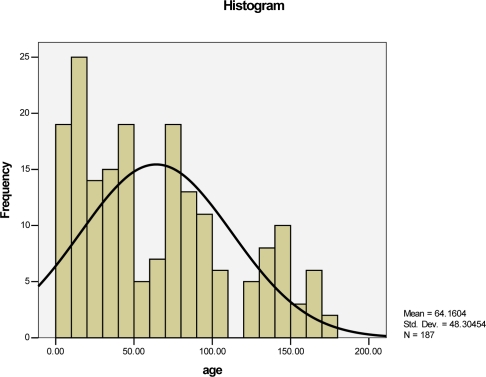
disrtribution of age in cases with acute pharyngitis.

100 cases were excluded due to the other diagnosis; 87 cases which had full inclusion criteria studied. Flow chart of cases is presented in Fig. 1.

Throat swabs obtained from 87 remaining cases. Immuno chromatograhic Rapid Test Strip(ACON; Germany)and pharyngeal culture on conventional blood agar were used simultaneously to detect the GABHS from 87 cases. Culture plates incubated in CO_2_ incubator for 18–24 hours. GABHS was identified as gram-positive cocci isolated from beta hemolytic colonies. Bacitrcin, SXT, Optochin, Biliary solubility; ClNa 6.5% tests were used for differentiation of GABHS (1).

Further complementary standard tests were usedfor dignosis of other organisms (*S. pneumoniae, N. meningitis, H. influenzae*)

We compared the results of two methods in cases. The Student's t test was used to determine significant differences in means for all continuous variables Chi square values (CI 95%, p<0.05) were calculated for all categorical variables. All analyses were conducted using SPSS13 software.

## RESULTS

The age of patients is showed in Fig. 1 and Table 1. 37.4% of cases aged between 1–4 years. Other signs and symptoms in cases included: Upper respiratory symptoms (46.3%),diarrehea (10%); vomiting (15%); abdominal pain (15%); conjunctivitis (7%); cervical/or submandibular lymph node (8.1%), exudate in pharynx (16%); petechia in pharynx (5.6%).

**Table 1:** Age groups in cases with acute pharyngitis.

Age group	Number	percent
<1 years	27	14.4%
1-4y	70	37.4%
5-9 y	56	30%
10-14 y	34	18.2%
Total	187	100%

**Fig. 2:**
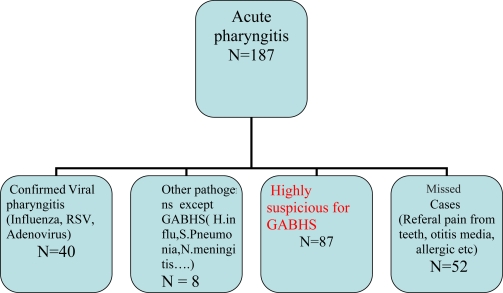
Flowchart of cases.

In 34.5% of pharyngitis cases, the GABHS antigens were detected by rapid test. Positive culture for group A Streptococcus obtained in 17.2% of pharyngeal culture. There was no agreement between two methods ( PV<0.1) (Fig. 2).

The cases with positive results by two methods (culture and rapid test) had mean age of 73 months with no differences

The cases with positive rapid test had mean age 73 months without significant difference with the cases with negative rapid test (mean age=60 month); p=0.4

Cases with positive rapid test was related to the age of the cases. (P<0.05)

The cases with positive culture had mean age 73 months which was different from cases with negative culture (90 months); p=0.059

Positive rapid test was related to observing the petechia in pharynx of patients (P<0.004).

Positive rapid test was not related to previous antibiotic treartment (p=0.1)

Results of culture was related to previous antibiotic treartment (pv=0.03)

Negative results in both methods are higher than expected in compare with other international references (16–18).

Negative pharyngeal culture observed in near half (43.2%) of the studied cases which might be due to the previous antibiotic treartment.

## DISCUSSION

Rapid antigen test and culture were positive in 34.5% and 17.2% of the cases respectively. There was no agreement between two methods (PV<0.1). Results by 2 methoeds are lower than recent reports (16, 17).

Negative results in both methods are higher than expected in compare with other international references (16–18). Negative culture in near half (43.2%) of the studied case might be due to the previous antibiotic treatment.

**Table 2:** Laboratory results in selected cases with acute pharyngitis.

percent	positive results/Studied cases	Lab results
34.5%	30/87	Positive *Rapid strip test*
17.2%	15/87	Positive GAS in throat culture
Pvalue=0/1; No agreement	87	Total

These results are very close to the previous studies in Iran. The carrier state for GABHS (9–12);and *S. pneumoniae* (13–15) in Iranian children are much lower (3-6%) than its rate in children living in developed countries (*S. pneumonia*: 21-59%) [1-3]. The high rate of Group G streptococci were reported in the recent Iranian studies (9–12). Frequent antibiotic treatment in Iran; exposure at different ages; different seasonalities could be the reasons for this wide differences.

According to Leung et al., most rapid antigen detection tests that are currently in use have an excellent specificity of greater than 95% and a sensitivity of greater than or equal to 90%. So, a positive rapid antigen detection test is accepted as adequate for the diagnosis of GABHS pharyngitis. Conversely, confirmation of a negative antigen detection test with a throat culture result is necessary (16).

The first RADTs used the latex agglutination technique. This is a relatively insensitive method with some-what unclear end points. Newer tests based on enzyme immunoassay (EIA) techniques offered more sharply defined end points as well as increased sensitivity (18).

Tanz et al reported 30% positive cultures. Rapid antigen-detection test sensitivity was 70% (17). Office culture sensitivity was significantly greater, 81% (range: 71%–91%). Rapid antigen-detection test specificity was 98%, and office culture specificity was 97%, a difference that was not statistically significant. They concluded that sensitivity of the office culture was significantly greater than the sensitivity of the rapid antigen-detection test, but neither test was highly sensitive (17).

Gerber et al compared the performance of various RADTs to each other or examined the performance of various RADTs in the office setting (18). The latest commercial RADTs for the diagnosis of GABHS pharyngitis to be developed are two tests that employ molecular biology methods which are expensive. Several investigations have demonstrated that this test has a sensitivity between 86 and 94.8% and a specificity between 95 and 100%. Currently a wide variety of RADTs, easy-to-perform are available for diagnosing GABHS pharyngitis (18).

## CONCLUSION

Rapid immunological GABHS antigen test in compare with conventional throat cultures, showed higher advantage for diagnosis especially in the cases with previous antibiotic usage. Presence of “ petechia in pharynx” was highly predictive of streptococcal pharyngitis in our cases. Diagnosis of streptococcal pharyngitis only based on clinical findings are misleading in most of the times.We prefer to add the rapid tests to pharyngeal culture for rapid diagnosis and treatment of streptococcal pharyngitis.
